# P-483. Epidemiology of Respiratory Viral Infections within the Neonatal Intensive Care Unit

**DOI:** 10.1093/ofid/ofaf695.698

**Published:** 2026-01-11

**Authors:** James E Fisher

**Affiliations:** Children's Hospital of Pittsburgh, Pittsburgh, PA

## Abstract

**Background:**

Respiratory viral infections continue to be a leading cause of hospitalization and morbidity in infants. Infection prevention programs focus on preventing the transmission of these viral pathogens within the inpatient hospital setting through the use of control measures. Viruses can be uniquely important in the neonatal intensive care unit (NICU) where many of the patients are particularly vulnerable. The specific make up of respiratory viral hospital acquired infections (HAI) within the NICU is not well established and the clinical impact of these viruses varies significantly.

**Table 1:**
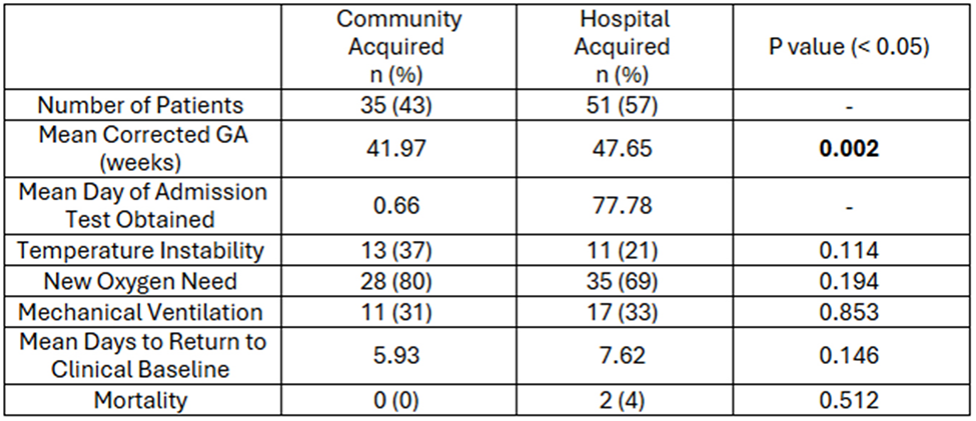
Clinical Symptoms and Outcomes of Community Acquired vs Hospital Acquired Infections

Clinical symptoms and demographics of patients with a positive test. Differentiated by community acquired (positive test or symptoms within 72 hours of admission) vs hospital acquired. All viruses were included in this analysis, though Rhino/enterovirus was the predominant pathogen observed. Outcomes between each group were similar, though the hospital acquired infection cohort had a

**Table 2:**
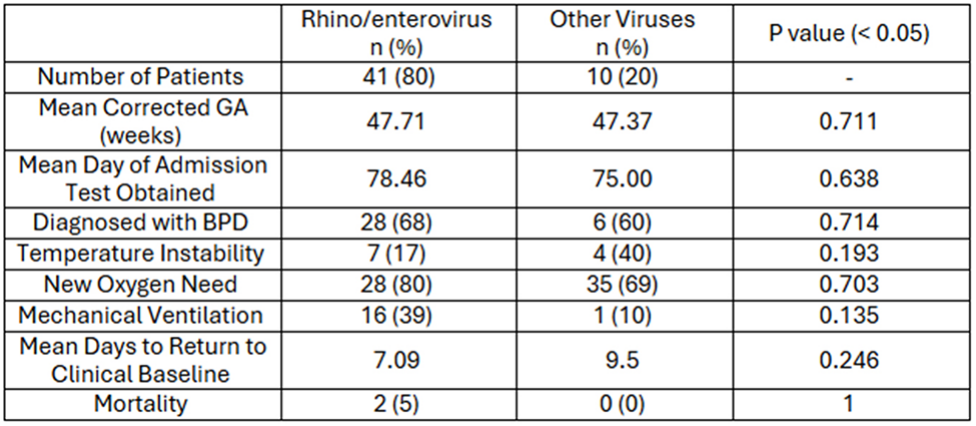
Clinical Course of Hospital Acquired Viral Infections Differentiated by Pathogen

Clinical symptoms between viral pathogens was comparable between rhino/enterovirus and other viruses observed. The rhino/enterovirus cohort was more predominant each year and comprised a majority of the HAIs observed. Though not statistically significant, there was a trend towards more frequent intubation in the rhino/enterovirus cohort.

**Methods:**

A retrospective chart review was performed on patients admitted to the NICU from 2022-2024 with a positive multiplex respiratory viral pathogen PCR panel (RVP). Clinical symptoms and associated morbidity or mortality was evaluated. The presence of viral infection clusters within the NICU was assessed based on timing and geographic definitions.

**Figure 1:**
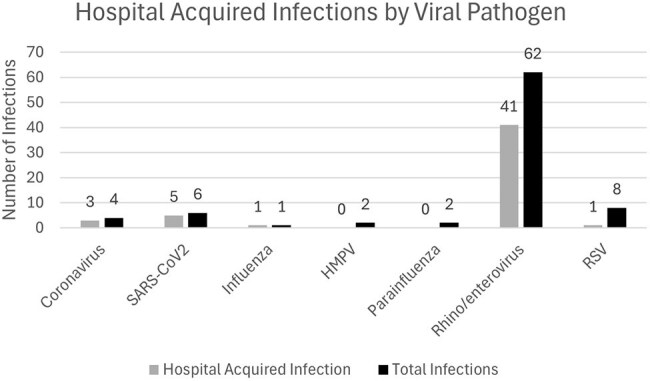
Hospital Acquired Infections by Viral Pathogen

Viruses identified on multiplex viral pathogen PCR panel separated by hospital acquired infection vs total virus samples identified. Rhino/enterovirus was the predominant pathogen and represented roughly half of the identified hospital acquired infections.

**Figure 2:**
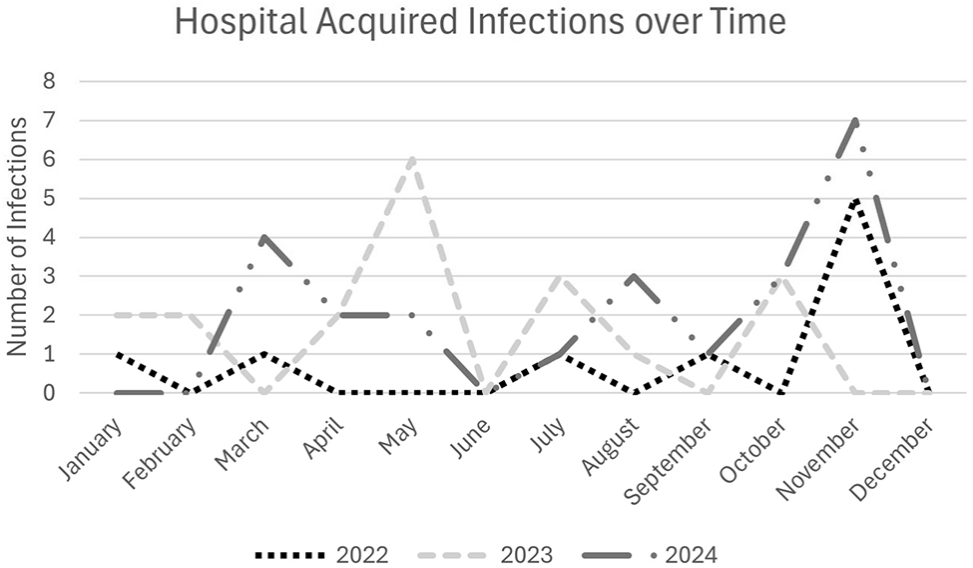
Hospital Acquired Infections over Time

Incidence of hospital acquired infections over the course of the year. An increase in cases was seen during the traditional respiratory viral seasons of Oct to Feb, but there were additional increases during summer months as well. November was the month with the most cases overall.

**Results:**

86 positive RVP samples were reviewed from 81 unique patients. 51 (59%) of the encounters were deemed to be HAIs based on timing and accepted National Healthcare Safety Network (NHSN) definitions. Multiple viruses were identified, but there was a predominance of rhino/enterovirus (78.7%) and SARS-COV2 (10.6%), with the remaining viruses being coronavirus, influenza, and RSV. Of the patients with associated HAIs, 69% had a new oxygen requirement and 33% required mechanical ventilation during their illness. The time to return to clinical baseline was roughly 7.6 days with this cohort. There were 2 (4.4%) associated deaths.

10 viral clusters were identified over the 3-year period with 2-5 patients being associated with each cluster. Rhino/enterovirus was the predominant virus, being associated with 90% of the identified clusters. 31 (60.8%) of the identified HAIs were a part of a viral cluster.

**Conclusion:**

Though commonly thought to be a benign virus we found that rhino/enterovirus infections within the NICU were associated with comparable morbidity. HAIs tended to present in clusters, though the mode of transmission between patients is unclear. HAIs were not isolated to the respiratory viral season and enhanced infection prevention measures may be needed throughout the year in high-risk units.

**Disclosures:**

All Authors: No reported disclosures

